# Hyperpolarized ^3^He magnetic resonance imaging ventilation defects in asthma: relationship to airway mechanics

**DOI:** 10.14814/phy2.12761

**Published:** 2016-04-06

**Authors:** Del Leary, Sarah Svenningsen, Fumin Guo, Swati Bhatawadekar, Grace Parraga, Geoffrey N. Maksym

**Affiliations:** ^1^ Department of Environmental and Radiological Health SciencesColorado State UniversityFort CollinsColorado; ^2^ Robarts Research InstituteThe University of Western OntarioLondonCanada; ^3^ Department of Medical BiophysicsThe University of Western OntarioLondonCanada; ^4^ Graduate Program in Biomedical EngineeringThe University of Western OntarioLondonCanada; ^5^ University Health Network‐Toronto Rehabilitation InstituteTorontoCanada; ^6^ School of Biomedical EngineeringDalhousie UniversityHalifaxCanada

**Keywords:** FEV_1_, heterogeneity, hyperpolarized MRI, lung impedance, ventilation defects

## Abstract

In patients with asthma, magnetic resonance imaging (MRI) provides direct measurements of regional ventilation heterogeneity, the etiology of which is not well‐understood, nor is the relationship of ventilation abnormalities with lung mechanics. In addition, respiratory resistance and reactance are often abnormal in asthmatics and the frequency dependence of respiratory resistance is thought to reflect ventilation heterogeneity. We acquired MRI ventilation defect maps, forced expiratory volume in one‐second (FEV_1_), and airways resistance (Raw) measurements, and used a computational airway model to explore the relationship of ventilation defect percent (VDP) with simulated measurements of respiratory system resistance (R_rs_) and reactance (X_rs_). MRI ventilation defect maps were experimentally acquired in 25 asthmatics before, during, and after methacholine challenge and these were nonrigidly coregistered to the airway tree model. Using the model coregistered to ventilation defect maps, we narrowed proximal (9th) and distal (14th) generation airways that were spatially related to the MRI ventilation defects. The relationships for VDP with Raw measured using plethysmography (*r* = 0.79), and model predictions of R_rs>14_ (*r* = 0.91, *P* < 0.0001) and R_rs>9_ (*r* = 0.88, *P* < 0.0001) were significantly stronger (*P* = 0.005; *P* = 0.03, respectively) than with FEV_1_ (*r* = −0.68, *P* = 0.0001). The slopes for the relationship of VDP with simulated lung mechanics measurements were different (*P* < 0.0001); among these, the slope for the VDP‐X_rs0.2_ relationship was largest, suggesting that VDP was dominated by peripheral airway heterogeneity in these patients. In conclusion, as a first step toward understanding potential links between lung mechanics and ventilation defects, impedance predictions were made using a computational airway tree model with simulated constriction of airways related to ventilation defects measured in mild‐moderate asthmatics.

## Introduction

Pulmonary imaging using inhaled gas magnetic resonance imaging (MRI) has previously revealed spatially and temporally persistent ventilation defects in chronic obstructive pulmonary disease (COPD) and asthma (de Lange et al. 1999, 2007; Altes et al. 2001; Samee et al. 2003; Parraga et al. 2007). While ventilation MRI is typically acquired in breath‐hold, ventilation defects may reflect the ventilation heterogeneity previously estimated using multibreath gas washout (Verbanck et al. 2003; Downie et al. 2007) and positron emission tomography (PET) imaging (Venegas and Musch 2005; Venegas et al. 2005) typically measured using tidal breathing techniques. The size and extent of MRI ventilation defects was previously evaluated in relation to the forced expiratory volume in 1 s (FEV_1_) and showed modest correlations in asthma (de Lange et al. 2006) and COPD (Kirby et al. 2010, 2011). In addition, previous work (Costella et al. 2012) also showed weak‐to‐no relationships for ^3^He MRI ventilation defect percent (VDP) and FEV_1_. Recently, we explored the relationship of MRI ventilation defects and abnormally remodeled airways in asthmatics with and without ventilation defects (Svenningsen et al. 2014), and observed that asthmatics with ventilation defects reported significantly greater inflammation, airways resistance, airflow limitation, and hyper‐responsiveness than asthmatics without defects. However, there are still many unanswered questions and some controversy about the role that biomechanical lung abnormalities may play in the temporally and spatially persistent ventilation abnormalities that are visibly and quantitatively obvious in asthmatics.

Pulmonary biomechanics can be estimated using the forced oscillation technique (FOT). Previous FOT studies showed that respiratory system resistance (R_rs_) and reactance (X_rs_) were sensitive to heterogeneous airway narrowing (Lutchen and Gillis 1997; Kaczka et al. 1999) and that X_rs_ was particularly sensitive to distal or peripheral airway narrowing (Kaczka et al. 1999). Modeling studies also showed that PET‐derived ventilation defects were correlated with respiratory system resistance measured at 0.15 Hz (Tgavalekos et al. 2005, 2007). By simulating bronchoconstriction in an asymmetric branching airway tree model, the relationship of ventilation defects with predictions of R_rs_ and X_rs_ at 6 Hz (Leary et al. 2014) were also shown. In other modeling studies, the distribution of narrowed airways responsible for generating ventilation defects was evaluated as was the relationship of ventilation defects with different frequency‐dependent patterns of impedance (Tgavalekos et al. 2005; Campana et al. 2009; Kaczka et al. 2011). The relationship of ventilation defects with respiratory impedance is not well understood; while the frequency dependence of R_rs_ (typically R_rs_ at 5 Hz minus R_rs_ at 20 Hz) and X_rs_ are sensitive to peripheral airway heterogeneity (Lutchen and Gillis 1997; Kaczka et al. 1999), their relative sensitivities, and the physiological relevance of this have not been determined.

To better understand the relationship of respiratory system mechanics and MRI ventilation defects, we coregistered MRI ventilation maps with a computational multibranch airway tree and simulated respiratory measurements after closing airways close to ventilation abnormalities. We explored the relationship of ventilation defects with R_rs_ and the frequency dependence of R_rs_ and X_rs_ as well as experimentally measured FEV_1_ and airways resistance (Raw). We hypothesized that lung impedance predictions could be simulated using a model that incorporated ventilation defects measured in asthma patients to help better understand these relationships.

## Methods

### Study design and subjects

We adapted a three‐dimensional airway tree consisting of 64,895 airways (M. Tawhai, U. Auckland), with 32,447 terminal airways (dimension distributions shown in Table 1), to generate an airway tree computational model. A full description of the model was previously provided (Tawhai et al. 2004); briefly, the airway tree was derived from a volume rendered lung X‐ray computed tomography (CT) acquired in a patient including the eighth‐generation airways with the remaining generations constructed using a volume filling algorithm (Tawhai et al. 2009).

**Table 1:** Distribution of airway branches and diameters

Generation *n*	Branches *n*	Terminal Branches *n*	d_mean_ (mm)	d_max_ (mm)	d_min_ (mm)	d_std_ (mm)
1	1	0	14.12	14.12	14.12	0.00
2	2	0	11.13	12.07	10.20	1.32
3	4	0	9.17	10.00	8.00	0.85
4	8	0	6.30	8.00	4.40	1.08
5	16	0	4.62	7.00	3.30	1.22
6	32	0	3.28	6.20	2.69	0.77
7	64	0	2.75	6.00	1.81	0.70
8	128	0	2.27	5.40	1.29	0.54
9	256	2	1.85	3.90	0.79	0.42
10	508	7	1.51	2.90	0.59	0.40
11	1002	56	1.23	2.59	0.50	0.37
12	1892	206	1.01	2.38	0.48	0.32
13	3372	685	0.85	2.23	0.47	0.27
14	5374	1722	0.74	2.01	0.46	0.23
15	7304	2947	0.67	1.85	0.44	0.19
16	8714	4265	0.63	1.70	0.40	0.16
17	8898	4965	0.60	1.57	0.37	0.14
18	7866	4646	0.58	1.39	0.35	0.12
19	6440	4059	0.57	1.24	0.33	0.11
20	4762	3047	0.56	1.09	0.27	0.09
21	3430	2252	0.54	0.96	0.28	0.08
22	2356	1690	0.53	0.85	0.27	0.07
23	1332	992	0.50	0.75	0.34	0.06
24	680	508	0.48	0.71	0.29	0.06
25	344	290	0.43	0.64	0.29	0.07
26	108	108	0.38	0.57	0.24	0.07

d_mean_, mean airway diameter; d_max_, maximum airway diameter; d_min_, minimum airway diameter; d_std_, standard deviation of airway diameter.

As previously described (Costella et al. 2012), participants between 18 and 60 years of age with diagnosis of asthma and FEV_1_ ≥60%, provided written informed consent to a protocol approved by a local research ethics board. During a single two‐hour visit, MRI and spirometry were performed at baseline, post‐methacholine (at the provocative concentration resulting in a 20% decrease in FEV_1_ (PC_20_) or the final dose) and 25 min after administration of 200 mg salbutamol through a pressurized metered dose inhaler (pMDI) and Aero Chamber Plus valve holding chamber (Trudell Medical International, London, Canada). Spirometry was performed using an ndd EasyOne spirometer (ndd Medizintechnik AG, Zurich, Switzerland). Plethysmography was performed 10 min prior to methacholine challenge using a MedGraphics Elite Series unit (MedGraphics, St. Paul, MN). Methacholine challenge was performed in the seated position according to ATS guidelines (Crapo et al. 2000) using an AeroEclipse II Breath Actuated Nebulizer (Trudell Medical International) until PC_20_ was achieved or a maximum dose of 16.0 mg/mL was administered.

### Image acquisition

Anatomical proton (^1^H) and hyperpolarized ^3^He MR images were acquired using a 3 Tesla Discovery MR750 system (General Electric Health Care; Milwaukee, WI), as previously described. Subjects (Parraga et al. 2007) were instructed to inhale a fixed 1 L gas mixture (N_2_ for ^1^H MRI and a ^3^He/N_2_ for ^3^He MRI) from functional residual capacity (FRC), and coronal images were acquired under breath‐hold conditions. It is important to note that as previously described, (Costella et al. 2012) the same volume of inhaled gas was used, regardless of FRC or TLC (Table 2). While the signal‐to‐noise ratio (SNR) was preserved (because we were not in the SNR‐limited range), the lung volume relative to TLC for image acquisition was slightly different for each patient. Scans at all time points were performed in the supine position and completed within five minutes of patient positioning in the scanner. Conventional ^1^H MRI was performed before ^3^He MRI using the whole‐body radiofrequency coil and a fast gradient‐recalled echo method (total data acquisition time = 12 sec; TR/TE/flip‐angle = 4.3 msec/1.0 msec/30°; FOV=40 × 40 cm; matrix=128 × 80 (zero‐padded to 128 × 128); partial echo percent = 62.5%; BW = 62.50 kHz; one excitation; 14 sections; section thickness, 15 mm; zero gap), as previously described.(Parraga et al. 2007) Hyperpolarized ^3^He MRI was enabled using a rigid linear bird‐cage transmit/receive chest coil (RAPID Biomedical GmbH, Wuerzburg, Germany) and ^3^He gas was polarized to 30–40% using a commercial spin‐exchange polarizer system (Polarean Inc, Durham, NC). ^3^He coronal static ventilation images were acquired using a fast gradient‐recalled echo method with partial echo (total data acquisition time = 10 sec; TR/TE/flip‐angle = 3.8 msec/1.0 msec/7°; FOV = 40 × 40 cm; matrix = 128 × 80 (zero‐padded to 128 × 128); partial echo percent = 62.5%; BW = 62.50 kHz; one excitation; 14 sections; section thickness, 15 mm; zero gap), as previously described (Parraga et al. 2007).

**Table 2:** Baseline demographic characteristics and pulmonary function measurements

Parameter (±SD)	Asthmatics (*n* = 25)
Age years	35 (11)
Male/Female	11/14
BMI kg/m^2^	26 (5)
FEV_1%pred_	84 (15)
FVC %_pred_	93 (11)
FEV_1_/FVC %	74 (11)
IC %_pred_	111 (15)
FRC %_pred_	92 (15)
RV %_pred_	113 (25)
TLC %_pred_	101 (9)
Raw %_pred_	126 (69)^a^
Gaw %_pred_	63 (38)^a^
PC_20 _mg/mL	6 (23)

SD, Standard Deviation; BMI, Body Mass Index; FEV_1_, Forced Expiratory Volume in 1s; %_pred_, Percent Predicted; FVC, Forced Vital Capacity; IC, Inspiratory Capacity; FRC, Functional Residual Capacity; RV, Residual Volume; TLC, Total Lung Capacity; Raw, Airway Resistance; Gaw, Airway Conductance; PC_20_, provocative concentration of methacholine sufficient to induce a 20% decrease in FEV.

a *n* = 24.

### Image analysis

**Figure 1:**
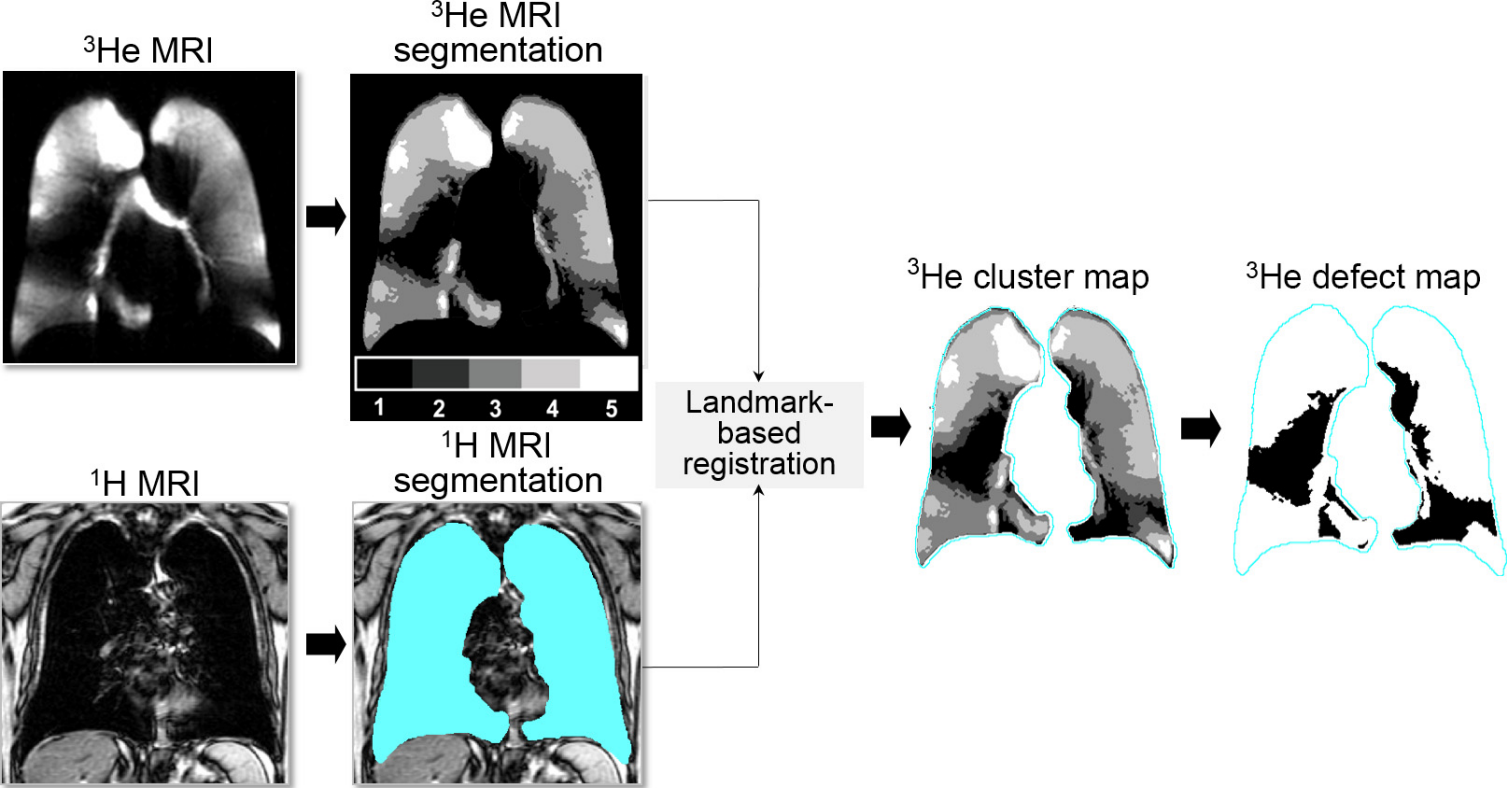
Pipeline for segmentation of ^3^He MRI ventilation defects. Raw image data from ^3^He MRI and conventional proton MRI are coregistered and a k‐means cluster algorithm was used to segment ventilation defects (shown as Cluster 1).

^3^He MRI semiautomated segmentation was performed using an algorithm implemented in MATLAB (The Mathworks Inc., Natick, MA), as previously described (Kirby et al. 2012). Briefly, and as shown in Figure 1, ^3^He MRI static ventilation images were segmented using a K‐means approach that classified voxel intensity values into five clusters ranging from signal void (cluster 1, C1 or ventilation defect volume [VDV]) and hypointense (or partial volume) to hyperintense signal (C5). The delineation of the ventilation defect boundaries was performed using a seeded region‐growing algorithm that segmented the ^1^H MRI thoracic cavity, as previously described (Kirby et al. 2012). This approach was previously validated using ventilation images in patients with asthma, cystic fibrosis, and COPD, and was based on a previous definition of ventilation defect volume as cluster 1 based on an expert visual interpretation of a series of images. Based on this previous information and numerous previous studies (Costella et al. 2012; Kirby et al. 2012; Svenningsen et al. 2014; Pike et al. 2015), we used this definition in this study.

### Coregistration of MRI ventilation maps to airway tree

**Figure 2:**
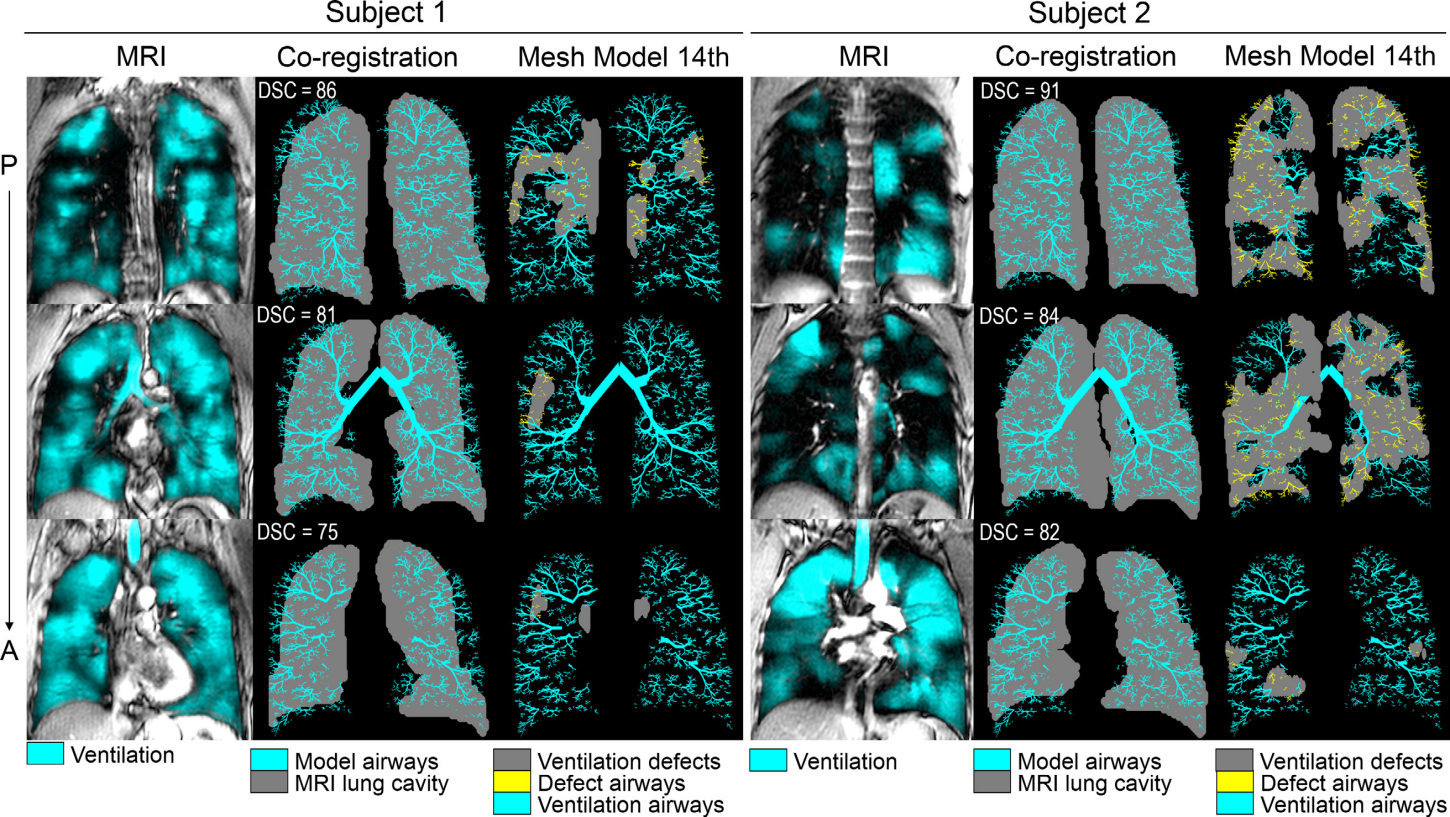
Coregistration of MRI and airway mesh model for three center slices in two representative asthmatics. For Subject 1 and 2, left panels show center, and 2 slices anterior (A) and posterior (P) to center coronal slice MRI; middle panels show corresponding model‐to‐MRI lung cavity coregistration; right panels show coregistration of model‐to‐ventilation defects coregistration with 14th generation airways proximal to ventilation defects closed in yellow. Slice‐specific Dice similarity coefficients (DSC) reflect registration accuracy for each of the three slices coregistered to the airway model for each of Subject 1 and 2.

We used rigid and nonrigid registration algorithms in MATLAB to coregister the MRI ventilation cluster map with the airway tree model. Briefly, the lung model was first resampled anterior‐to posterior into the same number of 15 mm slices as the coronal MRI datasets. A transformation matrix was generated so that each of the MRI coronal slices was rigidly coregistered to the mesh in the x, y, and z direction. Next, eight to 10 fiducial landmarks were manually located along the outer lung boundaries for each slice and using these fiducials, individual slices were rigidly coregistered to the mesh. This was performed for all datasets. It is also important to note that the rather coarse spatial resolution of the MR coronal slices was 3.13 mm_x_ × 3.13 mm_y_ × 15 mm_z_ and this places a conservative limit on registration accuracy. We note that we have routinely coregistered CT airway trees from individual patients to the MRI functional datasets in this manner (Pike et al. 2015) with fiducial localization error of 3–6 mm in the x and y plane and Dice similarity coefficients similar to what was achieved here (mean DSC = 83 ± 3%). In Figure 2, we show the coregistration results for three slices for each of the two subjects. The DSC ranged from 86 to 75% for Subject 1 and 91–82% for Subject 2, and this provides good evidence of coregistration accuracy. Figure 2 also shows the spatial relationship of 14th generation airways that were narrowed proximal to ventilation defects in these subjects.

### Impedance Predictions

**Figure 3:**
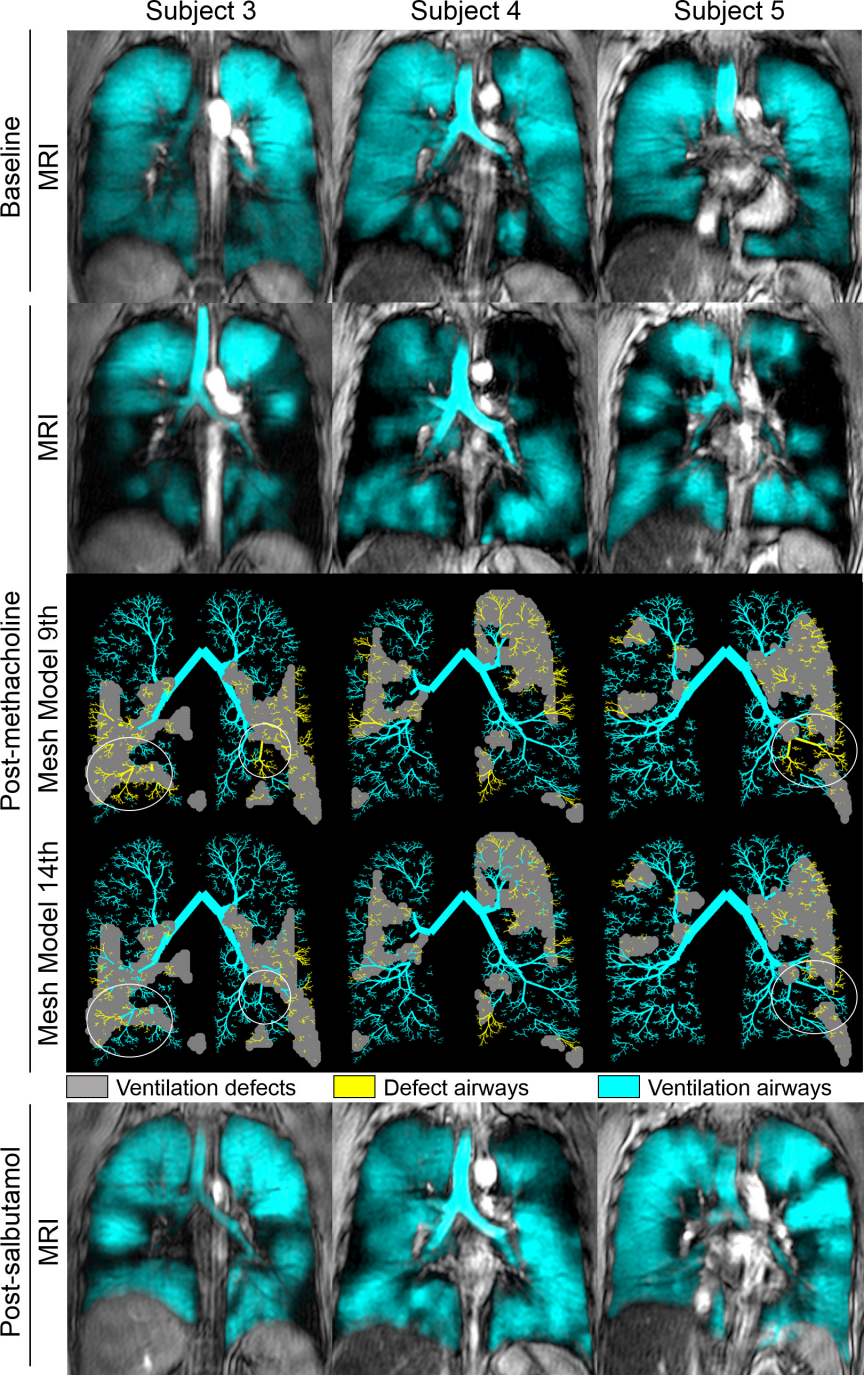
MRI and model‐to‐MRI coregistration for three asthmatics showing colocalization of airway tree and ventilation defects post‐methacholine. Center coronal slice MRI ventilation in blue coregistered to anatomical MRI in gray scale at baseline, post‐methacholine and post‐salbutamol. For each subject, the corresponding post‐methacholine mesh models with closure of airways from 9th (Row 3 across) or 14th generation (Row 4 across) are shown. For the mesh model, gray reflects an MRI ventilation defect, yellow segments are specific to mesh model airways located within a ventilation defect and cyan segments airways were mesh model airways that did NOT lead to ventilation defects. White circles show differences reflected by closure of either the 9th (row 3) or 14th (row 4) generation airways in the model. Subject 3: 36‐year‐old female; baseline/post‐methacholine/post‐salbutamol FEV_1_ = 66/45/71%_pred,_ VDP = 9/50/10%, R_rs>9_ = 2.97/4.64/3.12 cmH_2_O.s.L^−1^, R_rs>14_ = 2.71/3.54/2.78 cmH_2_O.s.L^−1^, X_rs>9_ = −2.20/−3.93/−2.16 cmH_2_O.s.L^−1^ and X_rs>14_=−1.64/−2.68/−1.49 cmH_2_O.s.L^−1^. Subject 4: 42 year‐old male; baseline/post‐methacholine/post‐salbutamol FEV_1_ = 72/50/77%_pred,_ VDP = 8/25/9%, R_rs>9_ = 2.71/4.08/3.04 cmH_2_O.s.L^−1^, R_rs>14_ = 2.67/3.21/2.76 cmH_2_O.s.L^−1^, X_rs>9_ = −1.63/−4.28/−2.35 cmH_2_O.s.L^−1^ and X_rs>14_ = −1.43/−2.51/−1.62 cmH_2_O.s.L^−1^. Subject 5: 46‐year‐old male; baseline/post‐methacholine/post‐salbutamol FEV_1_ = 60/43/62%_pred,_ VDP = 3/21/3%, R_rs>9_ = 2.72/3.94/2.73 cmH_2_O.s.L^−1^, R_rs>14_ = 2.64/3.22/2.63 cmH_2_O.s.L^−1^, X_rs>9_ = −1.46/−3.45/−1.37 cmH_2_O.s.L^−1^ and X_rs>14_ = −1.21/−2.03/−1.14 cmH_2_O.s.L^−1^.

Airways within ventilation defects (>10 voxels in the x or y direction (or 3 cm)) were narrowed to 10% of initial diameter as this effectively increased resistance by a factor of 10^4^ according to Poiseuille's law and avoided diameters of zero in the simulations (Tgavalekos et al. 2005). We occluded airways located within ventilation defects and within two voxels of their boundary at two different airway generations (9th or 14th generations) as shown in Figure 2 for two representative subjects, with airways proximal to ventilation defects shown in yellow and the ventilation abnormalities shown in gray. The rationale for choosing both 9th and 14th generation airways was based on previous work (Tgavalekos et al. 2005) that endeavored to explore simulations stemming from the medium and small airways. It is important to note that for trained image observers, it is quite straightforward to identify the specific airways leading to ventilation defects including the relatively large continuous spatial clusters >10 voxels we evaluated here. It is also worth pointing out that many ventilation defects were segmental and subsegmental defects as shown in Figures 2 and 3, and in these cases, the airway ventilation defect spatial concordance is also anatomically obvious.

Lung model impedance predictions were generated as previously described (Bhatawadekar et al. 2015) where the initial lung volume was reduced from TLC to FRC by reducing the airway diameters and lengths to 80%, achieving a volume ratio of 0.5. Flow was described by Womersley (Kaczka et al. 2007) where the complex impedance of each nonterminal airway branch was calculated as

(1) $$Z_{a}\left(f\right)=\frac{j2f\rho_{\mathrm{air}}l_{a}}{{r_{a}}^{2}}{\left[1-\frac{2J_{1}\left(\alpha_{a}\sqrt{-j}\right)}{\alpha_{a}\sqrt{-j}J_{0}\left(\alpha_{a}\sqrt{-j}\right)}\right]}^{-1}$$

where r_a_ is the radius and l_a_ is the length of the airway, f is the oscillation frequency in Hz, *ρ*_air_ is the density of air (1.16 kg/m^3^), J_0_ and J_1_ are the complex Bessel functions of order 0 and 1, respectively, j, the unit imaginary number, and *α*_a_ is the Womersley number of the airway branch given by

(2) $$\alpha_{a}=r_{a}\sqrt{\frac{2\Pi \rho_{\mathrm{air}}f}{\mu_{\mathrm{air}}}}$$

where *μ*_air_ is the dynamic viscosity of humid air at 37°C (1.85 × 10^−5^ Pa.s). Lung compliance was distributed evenly at terminal airways as each served as an alveolar compartment accounting for parenchymal stretch, surface tension, and gas compression (Tgavalekos et al. 2003). We recognize that this approach neglects the contributions of airway wall compliance and gas compression within airways, but this effect is much smaller than the effect of the alveolar compartment (Mead 1979; Leary et al. 2014). The impedance of each terminal airway was defined as

(3) $$Z_{t}=Z_{a}-j\frac{Et}{\omega }$$

where E_t_ is the elastance of the terminal airway unit. The model lung impedance (Z_L,mod_) was evaluated from series and parallel summations of all airway impedances assuming a lumped element approach, and separated into resistance (R_L,mod_) and reactance (X_L,mod_), and evaluated from 0.2 to 32 Hz.

Upper airway resistance (R_central_) and chest wall resistance (R_cw_) were assigned values of 0.5 cmH_2_O.s.L^−1^ each (Nagels et al. 1980; Barnas et al. 1987, 1989) and these were added to R_Lmod_ to obtain the model respiratory system resistance R_rs_. Due to the high‐frequency dependence of X_rs_, we also computed elastance which is largely frequency independent in healthy lung. Chest wall elastance (E_cw_ = 10.6 cmH_2_O.s.L^−1^) (Barnas et al. 1985) was summed with lung elastance (E_rs,mod_) to obtain the respiratory system elastance (E_rs_), where E_rs_ was 2.*π*.f.X_rs_(f). We computed E_rs_ for low frequencies only where X_rs_ was dominated by elastic mechanics. The frequency dependence of R_rs_ was evaluated over two ranges: 1) the low‐frequency range of 0.2–5 Hz (R_rs_0.2–R_rs_5) which is normally not accessible by common forced oscillation methods (but where the frequency dependence of R_rs_ is more easily observed), and, 2) the oscillometry frequency range of 5–20 Hz (R_rs_5–R_rs_20). Finally, the effects of upper airway shunt (Z_uaw_) were implemented using previously published Z_uaw_ values (Cauberghs and Van de Woestijne 1983) and extrapolated values of shunt resistance and reactance at 0.2 Hz as previously described (Bhatawadekar et al. 2015).

### Statistical analysis

Data were tested for normality using the Shapiro–Wilk normality test and nonparametric tests were performed when data were not normal. Univariate relationships were evaluated using linear regressions (r^2^), Pearson correlations (r), and Spearman correlations (*ρ*) when the data were not normal generated using GraphPad Prism version 4.00 (GraphPad Software, Inc., San Diego, CA). The Fisher z transformation was used to determine significant differences between r values. Significant differences in the VDP‐impedance relationship slopes were determined using analysis of covariance (ANCOVA). Holm–Bonferonni corrections were used for multiple comparisons. All results were considered statistically significant when the probability of making a Type 1 error was less than 5% (*P* < 0.05).

## Results

### Subjects

Table 2 provides subject demographic and pulmonary function measurements for 25 mild‐moderate participants with asthma (mean age 35 ± 11 years), moderately abnormal FEV_1_/FVC and airways resistance (Raw = 126 ± 69%_pred_). Figure 3 shows ventilation images (pre and post‐methacholine and post‐salbutamol) and model‐to‐MRI coregistration results (post‐methacholine) for three asthmatics. For all three subjects, methacholine induced a larger size and greater number of ventilation defects that partially resolved post‐salbutamol. The coregistered model‐to‐MRI results show the spatial localization of the 14th or 9th generation airways closed within ventilation defects and used to generate the simulated impedance measurements.

### Model and experimental measurements

**Figure 4:**
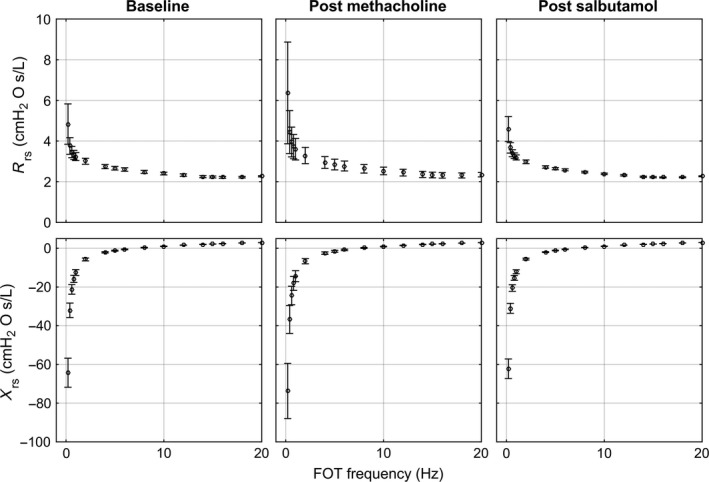
R_rs_ and X_rs_ versus frequency at baseline, post‐methacholine, and post‐salbutamol. Each point represents the mean value of the predictions for the 25 patients and the error bars represents the standard deviation of the distribution.

In Figure 4, the frequency‐dependent R_rs_ and X_rs_ predictions at baseline, post‐methacholine, and post‐salbutamol are provided. Table 3 shows measurements acquired and model predictions related to these three conditions. FEV_1_, VDP, and model predictions of R_rs_ and X_rs_ at 5 Hz were significantly different post‐methacholine and post‐salbutamol. Underscoring these significant differences, in Table 4, the fractional changes (baseline – post‐methacholine and post‐methacholine – post‐salbutamol) are shown for R_rs>14_ at different frequencies and frequency bands.

**Table 3:** Baseline, post‐methacholine and post‐salbutamol FEV_1_, VDP and model predictions

Parameter (±SD)	Baseline (*n* = 25)	Post‐methacholine (*n* = 25)	Post‐Salbutamol (*n* = 25)	P‐value^a^
Experimental				
FEV_1%pred_	84 (15)	64 (15)	87 (15)	<0.0001
VDP %	4 (4)	11 (10)	4 (2)	0.0002
Model				
R_rs>9_ cmH_2_O.s.L^−1^	1.99 (0.26)	2.33 (0.67)	1.92 (0.11)	0.003
R_rs>14_ cmH_2_O.s.L^−1^	1.85 (0.09)	2.00 (0.24)	1.84 (0.06)	0.002
X_rs>9_ cmH_2_O.s.L^−1^	−1.16 (0.34)	−1.54 (0.73)	−1.07 (0.18)	0.0008
X_rs>14_ cmH_2_O.s.L^−1^	−0.95 (0.13)	−1.14 (0.27)	−0.92 (0.09)	<0.0001

SD, Standard Deviation; FEV_1_, Forced Expiratory Volume in 1s; %_pred_, Percent Predicted; VDP, Ventilation Defect Percent; R_rs_, lung resistance; R_rs>9_, model prediction of R_rs_ when airways were closed distal to the 9th generation; R_rs>14_, model prediction of R_rs_ when airways were closed distal to the 14th generation; X_rs,_ lung reactance; X_rs>9_, model prediction of X_rs_ when airways were closed distal to the 9th generation; X_rs>14_, model prediction of X_rs_ when airways were closed distal to the 14th generation.

a Repeated measures analysis of variance.

**Table 4:** Fractional changes in VDP, FEV_1_ and model predictions post‐methacholine (relative to baseline) and post‐salbutamol (relative to post‐methacholine)

Parameter (±SD)	Post‐ methacholine/Baseline (*n* = 25)	Post‐Salbutamol/Post‐methacholine (*n* = 25)
Experimental		
FEV_1%pred_	0.76 (0.07)	1.37 (0.17)
VDP (%)	2.96 (1.50)	0.38 (0.20)
Model		
R_rs0.2_	1.31 (0.19)	0.77 (0.26)
R_rs5_ R_rs20_ R_rs0.2‐5_ R_rs5‐20_	1.07 (0.06) 1.03 (0.33) 1.25 (0.16) 1.56 (0.22)	0.94 (0.07) 0.97 (0.04) 0.07 (0.23) 0.45 (0.34)
X_rs0.2_	1.14 (0.08)	0.86 (0.11)
X_rs5_ X_rs20_	1.26 (0.10) 0.99 (0.01)	0.78 (0.16) 1.02 (0.01)

SD, Standard Deviation; FEV_1_, Forced Expiratory Volume in 1s; %_pred_, Percent Predicted; VDP, Ventilation Defect Percent; R_rs_, lung resistance; R_L0.2_, model prediction of R_rs_ measured at 0.2 Hz, R_rs5_, model prediction of R_rs_ measured at 5 Hz, R_L20_, model prediction of R_rs_ measured at 20 Hz, R_rs0.2‐5_, difference in model prediction of R_rs_ measured at 0.2–5 Hz, R_rs5‐20_, model prediction of R_rs_ measured at 20–5 Hz, X_L0.2_, model prediction of X_rs_ measured at 0.2 Hz, X_rs5_, model prediction of X_rs_ measured at 5 Hz, and X_rs20_, model prediction of X_rs_ measured at 20 Hz.

### Relationships for experimental measurements and model predictions

**Figure 5:**
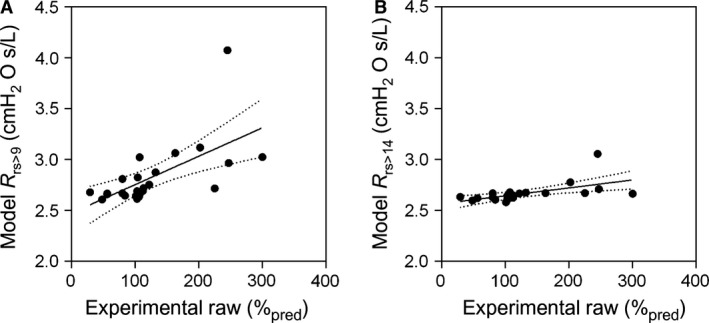
Relationship for Raw %_pred_ with model predictions of R_rs_ (cmH_2_O.s.L^−1^) at 5 Hz. Experimentally measured Raw %_pred_ using plethysmography at baseline compared with model predictions of R_rs_ based on airways distal to ventilation defects maximally closed at the A) 9th generation (*r* = 0.71, *r* ^2^ = 0.39, *P* < 0.0001, *y* = 0.003*x* + 2.47) and B) 14th (*r* = 0.71, *r* ^2^ = 0.34, *P* < 0.0001, *y* = 0.0008*x *+ 2.57) generation.

Table 5 shows correlation coefficients for FEV_1_%, Raw%, and model‐derived R_rs_ and X_rs_ at 5 Hz with VDP. There were moderate correlations for VDP with FEV_1_% and Raw% and significantly stronger correlations with R_rs_ and X_rs_. Some of these relationships are also shown in Figure 3 and 4. Figure 5 shows the relationship of Raw% measured before methacholine provocation with model predictions of R_rs_ when airways were closed from the 9th (*r* = 0.71, *P* < 0.0001) and 14th generation (*r* = 0.71, *P* = 0.0003).

**Table 5:** Relationships for FEV_1_, Raw, and model‐derived measurements at 5 Hz with Raw and VDP

	Raw %_pred_ (*n* = 24) r (*p*)	VDP % (*n* = 75) r (*p*)
Experimental		
FEV_1%pred_	−0.74 (<0.0001)	−0.68 (<0.0001)
Raw %_pred_	–	0.79 (<0.0001)^a^
Model		
R_rs>9_	0.71 (<0.0001)	0.88 (<0.0001)
R_rs>14_	0.71 (<0.0001)	0.91 (<0.0001)
X_rs>9_	–	−0.88 (<0.0001)
X_rs>14_	–	−0.94 (<0.0001)

Raw, Airway Resistance; VDP, Ventilation Defect Percent; FEV_1_, Forced Expiratory Volume in 1s; %_pred_, Percent Predicted; R_rs_, lung resistance; R_rs>9_, model prediction of R_rs_ when airways were closed distal to the 9th generation; R_rs>14_, model prediction of R_rs_ when airways were closed distal to the 14th generation; X_rs,_ lung reactance; X_rs>9_, model prediction of X_rs_ when airways were closed distal to the 9th generation; X_rs>14_, model prediction of X_rs_ when airways were closed distal to the 14th generation; *r* = Pearson r correlation coefficient.

a *n* = 24.

**Figure 6:**
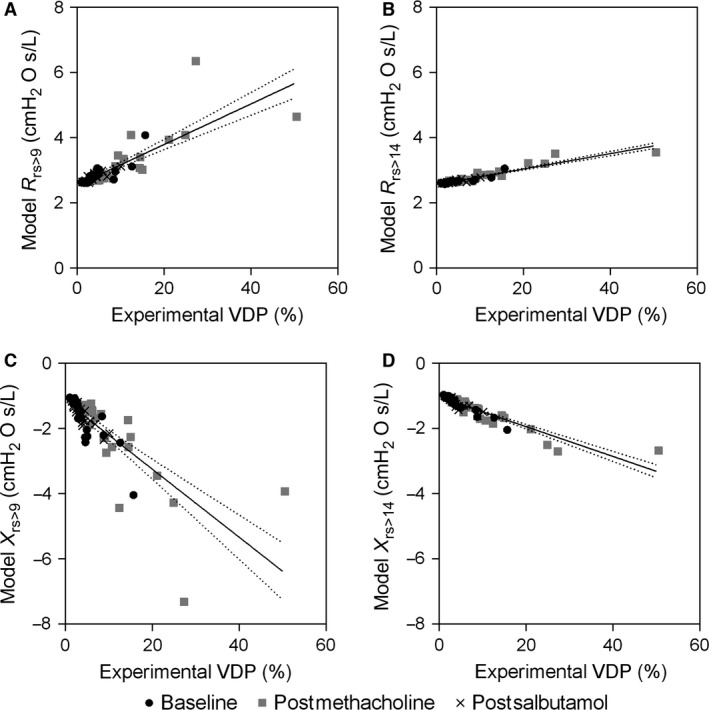
Relationship for ^3^He MRI VDP with model predictions of R_rs_ and X_rs_ at 5 Hz. There were significant relationships for VDP and model predictions of R_rs_ related to simulations of airways distal to ventilation defects maximally closed at the A) 9th (*r* = 0.88, *r* ^2^ = 0.67, *P* < 0.0001, *y* = 0.06*x *+ 2.54) and B) 14th (*r* = 0.91, *r* ^2^ = 0.89, *P* < 0.0001, *y* = 0.02*x *+ 2.56) generation, and model predictions of X_rs_ at the C) 9th (*r* = −0.88, *r* ^2^ = 0.60, *P* < 0.0001, *y* = −0.01*x* − 1.16) and D) 14th (*r* = −0.94, *r* ^2^ = 0.85, *P* < 0.0001, *y* = −0.05*x *− 1.03) generation.

As shown in Figure 6, R_rs_ was increased and X_rs_ at 5 Hz was diminished in relation to increasing (or worse) VDP. Figure 6A and B show impedance predictions at 5 Hz and the strong correlations between R_rs_ and VDP for 9th generation (*r* = 0.88, *P* < 0.0001) and 14th generation (*r* = 0.91, *P* < 0.0001) airway closures, respectively. Figure 6C and D also show strong correlations between X_rs_ at 5 Hz and VDP for 9th (*r* = −0.88, *P* < 0.0001) and 14th generation (*r* = −0.94, *P* < 0.0001) airway closures, respectively. The relationship between R_rs>14_ and VDP was significantly stronger than with FEV_1_ (*z* = 2.79, *P* = 0.005), but not with Raw (*z* = 1.86, *P* = 0.06). R_rs>9_ was more strongly correlated with VDP than with FEV_1_ (*z* = 2.24, *P* = 0.03), but not Raw (*z* = 1.31, *P* = 0.19). The correlation between X_rs>14_ and VDP was significantly stronger than the correlation between X_rs>9_ and VDP (*z* = 2.40, *P* = 0.02), whereas the relationships for R_rs>14_ and R_rs>9_ with VDP were not significantly different (*z* = 0.94, *P* = 0.34).

**Figure 7:**
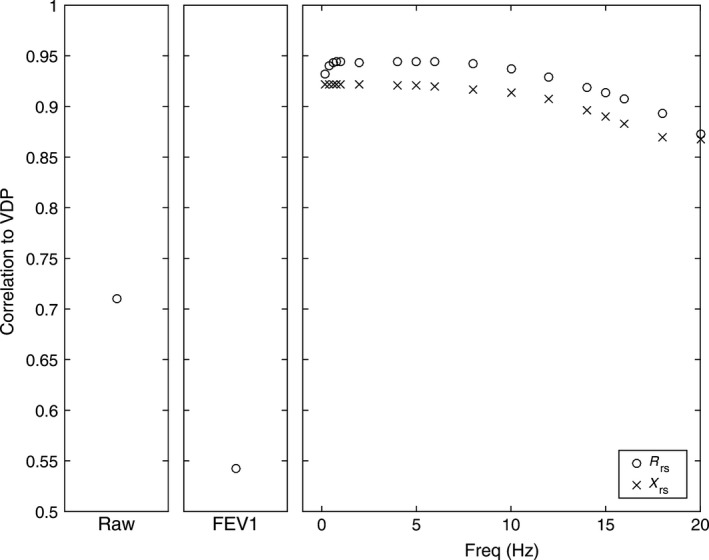
VDP correlations with experimentally measured Raw%_pred_ at baseline, FEV_1_%_pred_ and model predictions for R_rs_ and X_rs_ over a range of test frequencies.

**Figure 8:**
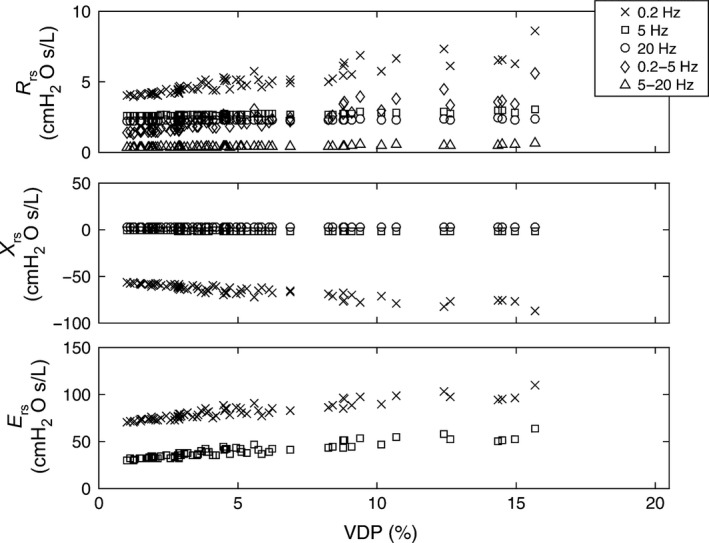
The sensitivity of measurements predicted by the lung model at various frequencies as indicated by the legend. The sensitivity to changes in VDP are indicated by increased slope (slopes for each frequency provided in Table 6). Measurements at lower frequencies showed greater dependence to VDP (i.e., X_rs_ and E_rs_ measured at 0.2 Hz reflected the greatest sensitivity to changes in VDP).

As shown in Figure 7, model predictions of R_rs_ and X_rs_ were strongly linearly correlated with VDP (at 5 Hz, R_rs_, *r* = 0.94, *P* < 0.0001; X_rs,_ *r* = 0.92, *P* < 0.0001), and these relationships weakened at greater frequencies. In Figure 8, the relationships for impedance measures with VDP are shown at specific frequencies, and the magnitude of these slopes are provided in Table 6. The slope that described the relationship for R_rs0.2_ and VDP was significantly greater than for the R_rs5_–VDP relationship (*P* < 0.001) and also significantly greater than the R_rs20_ –VDP relationship (*P* < 0.001). In a similar manner, the slope for the X_rs0.2_–VDP relationship was more negative than the X_rs5_–VDP relationship (*P* < 0.001). At all frequencies, the relationships between VDP and X_rs_ were stronger than those between VDP and R_rs_. For example, in the typical FOT range at 5 Hz, the X_rs5_–VDP relationship was stronger than R_rs5_–VDP (*P* < 0.001) and the R_rs5‐20_ –VDP (*P* < 0.001) relationships. The slopes for the relationship of E_rs_ with VDP are also apparently greater than the 5 Hz X_rs_–VDP relationship, but the larger slope is due to the change in units for E_rs_ to cmH_2_O.L^−1^ and not due to a stronger correlation.

**Table 6:** Relationships for model predictions with VDP

Model prediction	Slope
R_rs0.2_	0.223
R_rs5_	0.024
R_rs20_	0.009
R_rs0.2‐5_	0.199
R_rs5‐20_	0.015
X_rs0.2_	−1.356
X_rs5_	−0.046
X_rs20_	−0.005
E_rs0.2_	1.703
E_rs5_	1.435

## Discussion

We used ventilation defect maps acquired in asthmatics to constrain airway closure in a computational airway tree model and simulated measurements of airways resistance to investigate the influence of airway narrowing related to ventilation defects with predictions of lung biomechanical measurements and observed: (1) MRI ventilation measurements in 25 asthmatics acquired before, during, and after methacholine challenge could be used to close specific airways and drive simulations of lung biomechanical behavior, (2) model predictions showed greater mean R_rs_ and lower X_rs_ when specific airways were narrowed based on their proximity to MRI ventilation defects measured post‐methacholine, and (3) when ventilation defects were used to regionally constrain airway closure, the relationship between model predictions of X_rs_ and VDP was greater when more distal airways (14th vs. the 9th generation) were perturbed.

In asthma patients, ventilation defects increase in number and size during methacholine challenge and respiratory system resistance also increases (Tgavalekos et al. 2007). In a previous study, the relationship between PET‐derived ventilation defects and lung impedance was dependent on distal airway closure (Tgavalekos et al. 2005). Similar to this previous work, our model was constrained to physiologically relevant ventilation defects observed in a relatively wide variety of mild‐to‐moderate asthmatics. While it was not surprising that impedance increased with experimentally derived VDP, we were surprised that in asthma patients, this relationship was linear and statistically greater at the lower frequencies (in the range typically used by oscillometry). We also observed that when ventilation defects are used to regionally constrain airway closure, the relationship between model predictions of X_rs_ and VDP was greater when more distal airways (14th vs. 9th generation) were perturbed. While the physiological meaning of these findings is yet to be determined, as first demonstrated by Otis (Otis et al. 1956) using a single bifurcating airway, resistance and reactance are interdependent. In a homogeneous bifurcating airway tree, X_rs_ yields information related to the combined effect of the elastic and inertive properties of the lung where elastic effects dominate at low frequencies. In obstructive lung diseases like asthma, low‐frequency X_rs_, and thus E_rs_ can be strongly influenced by small airway narrowing, even, as our model suggested, when there are no changes in intrinsic tissue stiffness. This may reflect derecruitment of parenchyma whereby airway narrowing prevents distal oscillatory flow to the parenchyma diminishing lung elastance indicated by the more negative X_rs_. Although we observed the X_rs5_–VDP relationship was stronger in the distal airways, it is difficult to be certain about the physical meaning of this finding. It is important to note that we did not observe different R_rs5_–VDP correlations for the distal versus proximal airways. Taken together, however, these data suggest that R_rs5_ may be less sensitive than X_rs5_ and E_rs_ to conditions whereby the small airways are narrowed sufficiently to prevent oscillatory flow. Notably, previous work in pigs showed that R_rs_ was sensitive to airway narrowing and X_rs_ was more sensitive to ventilation defects (Dellaca et al. 2009).

The finding that X_rs5_ and E_rs_ predictions were related to VDP warrants further investigation and experimental confirmation in asthmatics. However, at 0.2 Hz, where the slope for the relationship between X_rs_ and VDP was the greatest, experimental confirmation will be challenging. Low‐frequency respiratory impedance can be determined using the optimal ventilation waveforms, but this approach is not amenable to clinical use. At the more common FOT frequencies, such as 5 Hz, model predictions of X_rs_ (and therefore E_rs_) were more sensitive to VDP than the frequency dependence of R_rs_ from 5 to 20 Hz. This sensitivity may arise from the peripheral airway narrowing which leads to alveolar derecruitment. However, the greater sensitivity of X_rs_ at 5 Hz compared to R_rs_ may be explained because resistance measurements at 5 Hz and 20 Hz include a large contribution from mainly unobstructed central airways. Low‐frequency X_rs_ and E_rs_ are dominated by the compliant parenchyma, with smaller contributions from the chest wall. This is in contrast to the small contribution of the peripheral airway relative to the upper and central airways to R_rs_, which may explain why airway narrowing in the periphery had a greater effect on X_rs_ than R_rs_.

Our study was limited and in that, we did not experimentally acquire oscillometry measurements; therefore, it is difficult to be certain about the physiological meaning of the relationships observed here between ventilation defects and respiratory resistance/reactance. Given the temporal nature of asthma provocation, it will be very difficult logistically to acquire these data experimentally within a methacholine challenge. Nonetheless, this modeling study cannot be considered definitive, but a hypothesis‐generating exploration of how an airway tree model can be used and constrained to help better understand resistance and reactance in asthmatics. Our model was limited and in that, we did not account for small degrees of constriction or dilation in the airways throughout the remaining lung volume – namely, the ventilated regions or central airways. This is important because lung regions outside of ventilation defects may be partially inflated or hyperinflated and this would alter R_rs_. We recognize that in asthmatics, ventilation defects are unlikely due to airway closure at specific generations. A possible future improvement in the model can be undertaken whereby more complex airway lumen sizing techniques at different generations can be used to scale airway diameter. It is also important to emphasize that we did not test how different spatial distributions of ventilation defects or patterns of bronchoconstriction influenced lung impedance. Therefore, we cannot comment on the sensitivity or specificity of impedance predictions to the spatial pattern of ventilation defects. However, despite normal variation in defect size and distribution among the subjects, we observed that experimentally measured Raw and predicted Rrs and Xrs at 5 Hz were strongly correlated with VDP. Also, we did not include upper airway narrowing in these simulations, but this could certainly lead to greater R_rs_ variability, relative to X_rs._

Ventilation heterogeneity is a hallmark finding in both asthma and COPD, but the biomechanical mechanisms responsible for ventilation defects in COPD and asthma may differ. In asthma patients, ventilation defects may be due to increased muscle activation acting on a normal parenchymal tethering load and/or mucous plugging (Downie et al. 2007). In COPD, there is the potential for diminished elastic recoil and thickened airway walls (Hogg et al. 2004). Indeed, in severe COPD, ventilation defects are regionally related to both emphysematous bullae and airway abnormalities (Kirby et al. 2014). In summary, we used an airway tree model to generate simulations of R_rs_ and X_rs_ based on ventilation defects in 25 asthmatics. Model predictions of low‐frequency X_rs_ and E_rs_ provide a way to explain airway behavior that may result in ventilation defects in asthmatics.

## Conflict of Interest

The authors declare they have no conflicts of interest to declare.
